# Stakeholders’ perceptions towards patients’ participation in promoting hand hygiene among health care workers in Wakiso district, Uganda

**DOI:** 10.1371/journal.pone.0312604

**Published:** 2024-10-24

**Authors:** Esther Buregyeya, Edwinah Atusingwize, Rebecca Nuwematsiko, Richard K. Mugambe, Tonny Ssekamatte, Ronald Tenywa, Fred Twinomugisha, Habib Yakub, Christine Moe

**Affiliations:** 1 Department of Disease Control and Environmental Health, Makerere University, School of Public Health, Kampala, Uganda; 2 The Centre for Global Safe Water, Sanitation and Hygiene, Rollins School of Public Health, Emory University, Atlanta, GA, United States of America; PLOS: Public Library of Science, UNITED KINGDOM OF GREAT BRITAIN AND NORTHERN IRELAND

## Abstract

**Introduction:**

Hand hygiene compliance is one of the key performance indicators for infection prevention and control programmes, patient safety and quality of health services. WHO guidelines and the patient centred approach stress the need to increase patient involvement in hand hygiene promotion in healthcare settings. Patients’ and health care workers’ perspectives are critical for developing interventions to foster patient involvement in promoting hand hygiene. This study explored perceptions of health care workers and patients towards patients’ involvement in hand hygiene promotion.

**Methods:**

An exploratory qualitative study was conducted in four health facilities: three public, and one private non-profit in central Uganda. We conducted key informant interviews (KIIs) with health care workers and focus group discussions (FGDs) with patients. Respondents were asked their views about a patient reminding a health care worker to practice hand hygiene and how best this can be done. Interviews were audio-recorded, and transcribed. Thematic content analysis was used.

**Results:**

We led seven FGDs grouped by sex (6 participants each), with patients from different units of the study health care facilities and 23 KIIs with the in-charges of the health care facilities, wards, and infection control committee members. The majority of the KIIs were in the age category 30 to 45 years (10/23), females (14/23), and 7/23 were nurses by cadre. For the FGD participants, the majority were aged 30 to 45 years (23/42), 24/42 were females, 21/42 had attained secondary education as their highest level of education and 21/42 were Catholics. The health care workers’ and patients’ views towards patients’ participation in promoting hand hygiene among health care workers are presented according to the four themes that emerged: i) Patients reminding health care workers to practice hand hygiene was offensive; ii) Patients fear of negative response from health care workers, including being denied or receiving poor quality services; iii) Role of management in influencing hand hygiene (patient reminding a health care workers to wash hands could be acceptable in private health facilities compared to the public ones); iv) Suggestions on how patients’ reminders to health care workers can be done, included empowering patients to do the reminders in a friendly and polite approach to the HCW.

**Conclusion:**

Patients are reluctant to remind health workers to practice hand hygiene, because they feel it is confrontational and embarrassing, while health care workers find it offensive. Patient involvement seems to threaten patient-provider relationships. However patient empowerment was reported to be critical in promoting it and this is in-line with the hand hygiene guidelines.

## Introduction

Clean, safe and well-equipped health care facilities are critical to the achievement of Sustainable Development Goals (SDGs)-SDG 3 focusing on good health and well-being, and SDG 6 on safe water and sanitation for all. Indeed, water, sanitation and hygiene (WASH) are a foundation of strong resilient health system [1]. Globally, the prevention and control of health acquired infections (HCAI) is an urgent issue, especially given the rise in antimicrobial resistance (AMR) [2,3]. The healthcare-associated pathogens that can lead to HCAI are transmitted through direct and indirect contact, with contaminated hands of healthcare workers being the most common and efficient vehicle of transmission in most settings [4]. Effective infection prevention and control (IPC) measures can thus bring multiple benefits: for prevention of HCAI including outbreaks of highly transmissible diseases and successful containment of AMR [5,6]. In particular, hand hygiene is recognized as the single most important practice to reduce HCAI and has also been associated with sustained decrease in the incidence of AMR infections in health care and community settings [4,5,7].

Hand hygiene compliance is a major safety and quality performance indicator of health care services [8]. The World Health Organization (WHO), recommends a hand hygiene compliance rate above 90% [9]. Though the importance of hand hygiene is well documented and recognized, heath care workers’ compliance is sub-optimal in many settings, with heath care workers washing their hands less often than recommended and using inadequate techniques [10–13].

Compliance with hand hygiene best practices during care of critically ill patients is only around 9% in low-income countries such as Uganda [11–13], and rarely exceeds 70% in high-income countries. This calls for additional efforts to improve the practice all over the world [14]. Addressing non-compliance with the guiding principles of hand hygiene requires more standardized policies, regular monitoring and surveillance, and additional research [15]. Evidence shows that multimodal programs/interventions have the greatest chance of increasing hand hygiene compliance and ensuring sustainable improvement [4,14,16]. In line with this the WHO Guidelines on hand hygiene among other strategies, encourage patients to promote and participate in hand hygiene. Partnerships are encouraged between patients, their families and health-care providers to promote hand hygiene in health care [4]. The guidelines also explicitly recommend the need for healthcare settings to foster a sense of empowerment in their patients [4].

Engaging patients in promoting hand hygiene is in line with the policy of patient—centered care approach, where involvement of patients and prioritizing their goals and values are critical, and at the heart of the care continuum [17]. The approach is gaining prominence in healthcare systems with its promise to increase patient satisfaction and improve health outcomes [17]. However, there is limited research on patient involvement in hand hygiene promotion, particularly in the low-income settings like Uganda. Therefore, we sought to explore the perceptions and attitudes of patients and health care workers towards patients’ role in promoting hand hygiene among health workers and to establish which approach they consider to be the most suitable for implementing an effective patient participation program to improve hand hygiene.

## Methods

### Study setting and design

The study was conducted in four health facilities: three public health care facilities (two health centre IVs and one healthcare centre III), and one private non-profit hospital in central Uganda. The selection of these facilities was purposive and aimed to include a private non-profit health care facility, and public/government-managed health care facilities at different levels (i.e., a hospital, health centre IV and healthcare centre III). This combination includes a representative range of health care facilities in terms of levels of healthcare provision and ownership/management in Uganda. The study used qualitative methods to explore the perceptions and attitudes of health care workers and patients towards patients’ participation in promoting hand hygiene among health care workers.

### Data collection

#### Participants

Data were collected between February and June 2021 among health care workers and patients. A total of seven focus group discussions (FGDs) and 23 key informants (KIIs) were conducted and this was determined by saturation level i.e., when no more new information came up. FGDs grouped by sex- to allow free sharing based on any gender considerations, were held with patients from different units of the study health care facilities who came in as outpatients. In total we conducted four FGDs with females and 3 FGDs with males. Each FGD comprised of six participants to allow for social distancing in the face of COVID-19. Participants for the FGDs were purposively selected based on who was present at the facility to seek care at the time of data collection. In addition, KIIs with the in-charges of the health care facilities, wards, and infection control committee members were conducted. Key informants were purposively selected based on their experience in providing care to patients, their role in promoting IPC at the health facility, and leadership position.

#### Data collection tools and procedures

Data was collected using FGD and KII guides. Separate interview guides for each category of respondents were developed. The FGD and KII guides were developed, based on the issue/topic to be explored i.e., perceptions and attitudes of patients and health care workers towards patients’ role in promoting hand hygiene among health workers. We also elicited which approach they considered to be the most suitable for implementing an effective patient participation program to improve hand hygiene. Semi-structured guides were developed with follow-on questions or probes were used accordingly. The KII guides were in English, and FGD guide was translated into the local language, Luganda. Both guides were pre-tested before the actual data collection exercise to check the relevancy and flow. Respondents were mainly asked about their views on a patient reminding a health care worker to practice hand hygiene, and suggestions on how best this could be done. Research assistants with experience in qualitative research selected participants and conducted the interviews. The research assistants received pre-training on the study objectives, research ethics and how to administer the FGD and KII guides. All the discussion with patients (FGD) were held in the local language, Luganda, while all KIIs were in English (although a few of them preferred to respond to some questions in the local language). All KIIs and FGDs were conducted in a place agreed upon with participants and deemed private, comfortable, and conducive for social distancing for the participants. Overall, interviews lasted between 45–60 minutes and were all audio-recorded (and notes taken) following a written informed consent.

### Data management and analysis

All audio files were transcribed verbatim in their original language of recording by the research team. Transcripts recorded in the local language were translated to English. All transcripts were printed, and hard copies reviewed independently by three people, EB, EA, and RN to familiarize themselves with the data. Data analysis was done following a thematic framework approach, based on the steps suggested by Braun and Clarke (2006). After reading selected transcripts several times, a code book was agreed upon and used to code all the transcripts manually. Related codes were merged into sub-themes, which later formed the themes. Analysis was mainly undertaken by (EB) supported by RN and RT. In case of disagreement, consensus was sought through discussion in a meeting where all the members of the analysis team attended. The authors discussed and reconciled the suggested codes and themes in the transcripts, see details in the results section.

### Ethical considerations

The study was approved by the Makerere University School of Public Health Higher Degrees Research and Ethics committee and the Uganda National Council of Science and Technology (HS817ES). Additional clearance was sought from the district-level health authorities, and directors of the selected health care facilities, and written informed consent was obtained from all participants. It was made clear to the participants that they have a right to discontinue the participation in the study at any time or to decline to answer questions that make them uncomfortable.

## Results

### Characteristics of study participants

Majority of the key informants were aged 30 to 45 years (10/23), female (14/23), and were nurses by cadre (7/23), Table 1. Most participants in the FGDs were also aged 30 to 45 years (23/42), female (24/42) and had attained secondary education as their highest level of education (21/42), while the majority were Catholics (20/42), Table 2.

**Table 1:** Socio-demographics of participants of the key informant interviews.

Variable	n (%), N = 23
**Age**	
24–29	6 (26.09)
30–45	10 (43.48)
above 45	7 (30.43)
**Sex**	
Male	9 (39.13)
Female	14 (60.87)
**Cadre**	
Nurse	7 (30.43)
Midwife	5 (21.74)
Clinical officer	4 (17.39)
Laboratory technician	4 (17.39)
Others	3 (13.04)
**Health facility**	
Private Not for Profit Hospital	9 (39.13)
1^st^ Public Health Facility, H/C IV	5 (21.74)
2^nd^ Public Health Facility, H/C IV	5 (21.74)
Public Health Facility H/C III	4 (17.39)

**Table 2:** Socio-demographics of participants of the focus group discussions.

Variable	n (%), N = 42
**Age** (**years**)	
19–29	12 (28.57)
30–45	23 (54.76)
Above 45	7 (16.67)
**Sex**	
Male	18 (42.86)
Female	24 (57.14)
**Highest level of education**	
None	4 (9.52)
Primary	13 (30.95)
Secondary	21 (50.00)
Tertiary	4 (9.52)
**Religion**	
Catholic	20 (47.62)
Anglican	8 (19.05)
Moslem	6 (14.29)
Pentecostal	6 (14.29)
Others	2 (4.76)

The health care workers’ and patients’ views are integrated and presented according to the four themes that were identified and illustrative quotes from respondents are used to exemplify the results. The four themes include i) Patients reminding health care workers to practice hand hygiene perceived to be offensive; ii) Patients fear of negative consequences from reminding health care workers to practice hand hygiene; iii) Role of management in influencing hand hygiene adherence; iv) Suggestions on how patients can participate in hand hygiene promotion among health care workers.

### Theme 1: Patients reminding health care workers to practice hand hygiene perceived to be offensive

The majority of health care workers and patients were negative to the idea of patients reminding health care workers to practice hand hygiene. Respondents generally felt that such reminders would not be acceptable as it was perceived that the health care workers are the custodians of knowledge and therefore difficult for a patient to tell them to practice hand hygiene when it should be the other way around. They added that such reminders from patients would imply that the health care worker is ignorant which could make him/her feel offended and embarrassed. They said that some health workers could develop poor attitudes towards a patient reminding them about the practice, thus damaging the patient-provider relationship.

*“That one may not be good…, it will show that the health worker is ignorant, and they may take it in a negative way. Though it is good, the health worker may take it negatively: that now you are the one teaching me? And yet he/she is the one supposed to teach the patient. It is like your child telling you, ‘Daddy, you have not washed your hands before eating’, he may be right, but you feel offended*.” **KII, In-charge Public Health Facility- HCIII (35 years)**

“*I don’t think that is good*, *because I don’t think it is the patients to remind you [health care worker] to wash hands … aah*, *no*, *that one is not a good one… It may lead to development of poor attitude*…*because if a patient tells you to wash hands*, *then you are like no*, *the patient has offended me*.” **KII, ANC In-charge Private Hospital (32 years)**

*“It is not easy*, *you cannot tell the health worker what to do*, *the health workers are rude*. *I don’t know why; you may tell a health worker something simple and you will see someone quarrelling”*. **FGD Female patients, Public Health Facility—HCIV**

Some respondents perceived the reminders as being unrealistic and demotivating amidst challenges of no resources especially by those health workers that are demotivated by the system’s challenges. Such feelings were thought that they would be strong especially for health care workers working in public/government health facilities where scarcity of resources including hand hygiene facilities and supplies were common. However, a few of the participants felt differently and were positive about patients promoting hand hygiene practices among health care workers. Key informants generally emphasized that the patient reminding a health care worker to practice hand hygiene would be received negatively by majority of the health care workers. However, a few health care workers indicated that patient reminders to practice hand hygiene would be perceived positively, as elaborated below.

“*Now that one (a patient reminding me) is very good for me as me. For me, if a patient reminds me, I take it positively. If a patient reminds me that ‘doctor, you did not wash your hands?’, I will do it***…,** *so I advise the health workers to take it positively and go and wash, if you have a sanitiser there, you sanitise!***”** *But I have some colleagues I know who can take it easy, but others will react negatively, and you know that there will be fire; some may even refuse to work on the patient*.” **KII, In-charge Public Health Facility—HCIV (53 years)**

### Theme 2: Patients fear of negative consequences from reminding health care workers to practice hand hygiene

Respondents were concerned about the potential consequences from the patients’ reminders to the health care workers regarding hand hygiene. Both patients and health care workers in this study mentioned that patients would hesitate to remind health care workers to practice hand hygiene due to the worry about related negative consequences including denial of health care or being offered poor services. In relation to this, a key informant mentioned that some patients raise their concerns about other inadequate health services they receive as they leave the facility, because of fear of being offered poor services or care if they complained while still getting the service. Participants reported that patients fear health care workers and therefore could not risk reminding them to practice hand hygiene.

“*I don’t think something might happen but what they fear most, ‘if I remind this health worker [about] what to do and yet I am still getting service from this person I may not get good service, let me keep quiet’. Some of them [instead] raise their concerns at the exit when it is already too late, or they raise the concern after some time.’***” KII, OPD In-charge Private Hospital (31 years)**

“*When you remind the health workers*, *they might even give you a wrong dose of medicine*, *so people fear to remind them*.” **FGD male patients, Public Health Facility—HCIV**

### Theme 3: Role of healthcare facility management in influencing hand hygiene adherence

Although, the idea of patients reminding health workers to practice hand hygiene was generally perceived as strange and not in order in both public and private health facilities, some respondents from the private health facility felt that patients have a right to demand for quality care because they pay for the services. It was reported that observation of infection control measures including hand hygiene is an important aspect of customer care which is key in the private health facility. The different acceptability (of patient’s reminders to healthcare on hand hygiene) between the private and public health facilities was therefore related to the nature of healthcare facility management that set different expectations and consequences. In the private facility, patients reminding health care workers was perceived as okay, although rare. It was noted that such reminders would be more acceptable compared to patients reporting/ complaining to the facility management where the consequences would be worse for the health care worker (than the embarrassment of a patient telling them to practice hand hygiene).

“*I have never seen anyone of our clients reminding our health workers to wash their hands… Of course, if a client reminds you to wash your hands you are supposed to comply…There is not any shame, in fact you should thank that person, because if that client goes and reports you to the administration up there, it is a different case*… *In fact, health care workers know the consequences. Because once it reaches up there [meaning to the management] it is different, not just what we are looking at down here. Infection control is key and is part of good customer care.*” **KII, In-charge HIV Clinic Private Hospital (34 years)**

In relationship to management, hand hygiene and acceptability of the reminders from the patients in private facilities, was specifically related to clients’ satisfaction which can affect the clients and business. Therefore, we noted that the patient reminders for health care workers on their hand hygiene would be responded to positively by the health care workers and the management in order to attract and maintain the clientele and business in private health facilities. Most participants indicated that such a management approach was less likely to be taken in the public health facilities where patients were perceived to have no right to complain about the services that they were not paying for.

*‘If one client is being dissatisfied to your service, is he or she going to keep quiet? And if he is not going to keep quiet, he is going to tell another one that at [names the facility] they do this and this. So, it is going to demotivate others from coming here, so the hospital does not look at it lightly as infection control, but they look at it in a bigger spectrum of losing a number of their clients. So, at the end they say the revenue we collect there is what is used pay your salary. So, if they did not collect the revenue the health worker should miss his/her salary… It is a good idea, but it depends how someone else will take it. These days health workers are very sensitive. Here even if you are annoyed you are supposed to work on that patient, but in public service, the next time you will not get service*.” **KII, In-charge HIV Clinic Private Hospital (34 years)**

“*It may not be easy for the patient to mention that [remind health care worker] in a public facility unlike the private sector where they know they are paying for the services*, *and they have a right to complain*.” **KII, OPD In-charge Private Hospital (31 years)**

“*That is a little challenging because it might work here but not in public sector because here being private setting*, *there is a way we take client*. *So*, *if a client told someone like that*, *we have people with different attitudes and characters*, *so the message might land on somebody who is going to take it positively*, *they will be ok with it*. *But if it lands on somebody who is going to take it personal*, *it would also bring problems*.*”* **KI, Maternity In-charge Private Hospital (37 years)**

### Theme 4: Suggestions on how patients can participate in hand hygiene promotion among health care workers

Some respondents made recommendations on how best the patients can participate in promoting hand hygiene among health care workers. While it was generally not seen as acceptable for the patients to remind health care workers to practice hand hygiene, some respondents felt that it could be okay depending on the approach used. In particular, it was noted that if the health care workers would be approached in a friendly and polite way, they would not take it badly.

“*It depends, because you may find that depending on the way this person also approaches you, it can be easy to deal with. But how you approach the person you are telling to do something matters a lot! The mood you are in as you tell that person matters a lot! I have said, it depends on how this someone has approached you, so if this patient has approached the health worker in a friendly way, they will react in a friendly way”* **KII, In-charge Public Health Facility—HCIII (35 years)**

Besides the approach, the context or situation within which the health care is provided was reported as a major factor in how the reminders from patients would be received by the health care workers. In particular, it was noted that work conditions including stress of health care workers plays a key role in how they would respond to such reminders by their patients.

“*But if you find that the health worker has come with his own stress and you are like, ‘musawo [health worker in local language], you have not washed hands’. I [health care worker] will be like; ‘what are you talking about? I know I am supposed to do it!’ It can be a bit hard but if this person comes in, in a gentle way and say, musawo how are you? and you respond, ‘I am fine’, and you say; Oh, musawo you have forgotten to wash hands. In a polite way. It can easily be done*.” **KII, In-charge Public Health Facility—HCIII (35 years)**

The need to empower patients to promote hand hygiene was also highlighted. Related to the fact that the health care workers were regarded as authority and custodians of knowledge, it was noted that patients inherently feared to remind them to do what would be the right thing (hand washing]. However, it was mentioned that if patients were empowered by for example, giving them knowledge about the importance of hand hygiene, they could be able to remind the health workers in an acceptable way, thus effectively participate in promoting hand hygiene.

“*The patients here fear to tell the health workers. But giving them the knowledge is good, they can be empowered that before the health worker does this, he is supposed to do this, such that they know. Maybe they can know how to pass on the information in a more polite way,… the way you pass on the message also matters*, … *It should be in a friendly way not to offend the health worker*.” **KII, In-charge Public Health facility—HCIII (35 years)**

In addition, the need to sensitize both the patients and health care workers was reported to be necessary for the implementation of the practice of patients promoting hand hygiene promotion among health workers. The sensitization of patients about their responsibility in promoting hand hygiene was thought to be a very critical component of building their knowledge and capacity to participate given that most of them have low levels of education.

**“***You first sensitize the patients how to deal with this. You know these are local people here, the ones we are dealing with, and I am sorry to say but most of them are not educated and it is very hard to deal with people who are not educated. You tell him something and he picks it in a wrong way, or he may also tell you something which intimidates you. So, sensitization on both sides (patients and health workers, then it can easily be worked on*.” **KII, In-charge Public Health Facility—HCIII (35 years)**

Some of the recommended approaches to ensuring patients’ participation in promoting hand hygiene in health facilities included use of ways and practices in which the patient would indirectly influence the health care workers’ hand washing. Some of the health care workers said that the patient could for example, request to wash his/her hands; an action that would likely tickle or remind the health care workers to wash his/her own hands as well. It was perceived that a health worker would practice hand washing in order not to be seen as operating below the hand hygiene standard set by the patient.

“*If I was the patient, instead of telling the health worker to wash hands, I will say that musawo [meaning health care worker], I would like to wash my hands. It will click in the health worker’s minds that if the patient wants to wash his/her hands, what about me? But telling her directly that wash your hands, you can be boxed [laughter], the health workers are tired*.” **KII, Maternity In-charge Public Health Facility—HCIV (47 years)**

## Discussion

This study assessed the perceptions of patients and health care workers regarding patient participation in promoting hand hygiene among the latter as well as establish which method they considered to be the most suitable for implementing the same. The findings revealed that; generally, health care workers and patients had negative perceptions on the idea of patients reminding health care workers to practice hand hygiene. Such reminders were perceived by respondents as not acceptable and could damage the patient and health care worker relationship consequently negatively impacting the quality of services the patients would receive. However, a few of the health care workers felt differently, with some reporting that they would be positive about patients reminding them to practice hand hygiene. For the patients, the fear of negative consequences including being denied services or being offered poor quality services was a big deterrent for them to remind health care workers to practice hand hygiene. However, the idea was seen as easily applicable only in a private health care facility setting where patients pay for the services, and were believed to have rights to demand for quality services as part of the expected customer care. Rather than face the negative repercussions in case the patient reported to management, private health care providers would rather bear the embarrassment of the patient reminding them to practice hand hygiene. Suggestions were made on how to ensure that reminders from patients would be acceptable.

Patients and health care workers indicated that patients’ reminding health care workers to practice hand hygiene is offensive because health care workers feel they are superior/custodians of knowledge. This is similar to what was reported in other studies [18] in UK and Jordan where both health care workers and patients reported concerns that asking about hand hygiene was offensive, embarrassing and upsetting to health care workers and could have an adverse impact on the health care worker-patient relationship. Similar findings were also reported elsewhere[19–21]. In addition, a review by Nacioglu on [22] on assessing the effectiveness of speaking up for patient safety, considering both patient and provider perspectives found that if a patient speaks up, it might result into unintended consequences. Speaking up was seen to be rude or disrespectful, making staff upset and endangering patient care. Therefore, though patient participation in hand hygiene might be appropriate when patients notice that health care workers do not wash or sanitise their hands before touching them, they may not have the courage to speak up to improve the quality and safety of their healthcare [22]. This is however contrary to the patient—centered approach which encourages collaboration and shared decision-making between providers, patients, and families. Patients and their families are expected to be part of the care team and should play a role in decisions at the patient and system level [23]. Therefore, this demands that the patient should be a partner and co-designer in his/her care and management, a concept that many health care workers still find threatening or unnecessary [24].

Our findings generally suggest that patients being actively involved in hand hygiene is not supported. Respondents felt that it would create conflict because of a judgmental perspective and lack of trust in health care workers it creates about them in delivering safe care [20,25]. The feeling of apprehension among patients in relation to telling health care workers to wash their hands was also reported elsewhere [25,26]. Patients might feel that this is not their responsibility [27] but also think that health care workers have enough expertise to recognize the importance of standard procedures in HCAI prevention without having patients to raise the subject [28,29]. Furthermore, the concept is also challenged by culturally engrained barriers in professional–patient relationships, such as power imbalances and clinical dominance where it may not be acceptable for patients to raise complaints to the health care worker who is regarded as most knowledgeable in their field [20,27,30]. Relatedly, in the Ugandan setting patient reminders are felt to be unacceptable and can cause confrontation from health care workers and affect quality of care. Patients reminding health care workers to practice hand hygiene is reported as a behaviour contrary to the social standards [31,32]. There is however evidence on variations across settings. A study conducted in Taiwan on patient empowerment in hand hygiene, reported that over 60% of patients had positive attitude to remind health care workers to wash their hands and similarly, health care workers were positive about being reminded [33]. Furthermore, Australian patients were reported to feel comfortable and happy to ask health care workers to wash their hands [21]. However, patients were more likely to be willing to ask a health care worker a factual question than a challenging question [30]. In two reviews conducted by McGuckin and Govednik [34] exploring empowerment and barriers to empowerment in hand hygiene and by Alzyood et al. [35] on perceptions of patients and healthcare professionals towards patient involvement in promoting hand hygiene, patients were found to be more inclined to ask nurses, rather than doctors, about their hand hygiene. In addition, findings from four studies carried out in the US [28,36–38] reported that knowing the health care worker’s name increases patient willingness to ask about hand hygiene. On the other hand, patients from Switzerland reported feeling uncomfortable in asking the health care workers wash their hands [39]. Similarly, a study by Kim et al [40] in South Korean reported that it is not the patient’s responsibility to remind health care workers to wash their hands.

We found that patients reminding health care workers to wash their hands is more applicable in a private health facility on the grounds that patients pay for the services and thus believed to have a right to demand for quality services. To our knowledge, this seems to be a new finding only reported in this study. Relatedly, although overall low, evidence shows that compliance with hand hygiene was better in private than public facilities in both newborn units and labour rooms in India [41]. Similarly, research from sub-Saharan Africa has shown consistently lower access to water, sanitation and hygiene services in public compared to private facilities [42], including the greater Kampala metropolitan area in Uganda [43]. Our finding that indicates WASH as a component of client satisfaction by the private facilities could be related with evidence that shows association of poor WASH provision with significant patient dissatisfaction with infrastructure and quality of care [44] which may influence patient choices and access to health facilities. For instance, a study among mothers living in hospital catchments, in Rukungiri and Kanungu districts in Uganda indicated that perceived higher quality of WASH services was one of the factors associated with delivery in a private facility compared to a public facility [45].

Respondents further suggested ways in which it could be made easy for both patients and health care workers to actively participate and promote hand hygiene in health care facilities. Recommendations were related to the approaches to be used to remind the health care workers and the need to empower patients to promote hand hygiene. Overall, the need to empower patients by giving them knowledge about hand hygiene and their related roles, and the acceptable polite and friendly approaches towards health care workers were highlighted. This is consistent with previous findings [18], and one of the strategic goals for implementing patient—centered care [46]. Previous evidence by McGuckin and Govednik [34] confirm that one cannot participate, be involved, or be engaged without the components of empowerment including knowledge, skills, and an accepting environment. This health information to be understood by patients, should be easily accessible to people with low levels of health literacy [47]. Patient education including watching learning videos, and providing them with authorization (such as the ‘It’s OK to Ask campaign’) have been some of the interventions responsible for an increase in patient intention to request that health care workers in UK wash their hands [34]. This concept of asking patients to remind health care workers to wash their hands may seem simple enough [25]; however, patient involvement in this process remains not fully explored especially in the low-income settings like Uganda.

To our knowledge this is the first study in Uganda to explore views on patients’ participation in promoting hand hygiene among health care workers. More research is needed to develop interventions that can increase and sustain patient involvement in promoting hand hygiene promotion as an essential component of patient safety in both private and public health facilities. Quantitative studies are necessary to enable statistical categorization and associations of health care workers’ hand hygiene behaviours and different factors (at individual and health system level). Further qualitative studies could provide more in-depth understanding into the enablers and barriers as well as appropriate approaches and theories that can enhance hand hygiene of health workers and patients themselves.

### Strengths and limitations of the study

To our knowledge this is the first study in Uganda to explore views on patients’ participation in promoting hand hygiene among health care workers, a component of multimodal strategy for hand hygiene that is not prioritized. This being qualitative research, we recommend for future larger quantitative studies amongst health care workers and patients from different levels of service delivery and facility ownership. More research is needed to inform development of interventions that can increase and sustain patient involvement in hand hygiene promotion as an essential component of patient safety in both private and public health facilities.

## Conclusion

In general patients’ participation in promoting hand hygiene among health care workers by reminding them to wash their hands was perceived as offensive, embarrassing and could strain patient and health care workers relationship. This is contrary to the recommended patient involvement in ensuring patient safety through the promotion of hand hygiene compliance. Empowerment of patients on their rights, roles and importance of hand hygiene and efforts to sensitize health care workers about patient involvement are important. More research is needed to understand situations in which patients would feel empowered enough to speak up and thus help develop interventions that can increase and sustain patient involvement in hand hygiene promotion as an essential component of patient safety in both private and public health facilities.
